# Traumatic life events and risk for dementia: a systematic review and meta-analysis

**DOI:** 10.1186/s12877-023-04287-1

**Published:** 2023-09-22

**Authors:** Emilia Severs, Tiffeny James, Pilar Letrondo, Lise Løvland, Natalie L. Marchant, Naaheed Mukadam

**Affiliations:** Division of Psychiatry, UCL, 6th Floor Maple House, 149 Tottenham Court Road, London, W1T 7NF United Kingdom

**Keywords:** Dementia, Risk, Traumatic life events

## Abstract

**Objectives:**

To systematically review the association between traumatic life events (TLE) and dementia risk.

**Design:**

Systematic review and meta-analysis.

**Data sources:**

APA, PsychINFO, Embase and MEDLINE from their inception to 29.05.21 and updated on 20.04.22.

**Eligibility criteria for selecting studies:**

Original research articles published in peer reviewed journals examining the association between TLE and all cause dementia in individuals aged 60 and over. Two researchers independently assessed the risk of bias using the Newcastle–Ottawa Scale. We conducted a generic inverse variance random effects meta-analysis to provide an overall estimate of TLE impact on dementia risk.

**Main outcome measures:**

Risk, odds and hazards ratios relating to dementia risk.

**Results:**

Initially, 3,487 studies were retrieved in the search and seven studies were included in the meta-analysis with data being used from 276,570 participants. TLE were associated with increased dementia risk. Trauma in general had a pooled HR of 1.21, (95% CI 1.03, 1.43, P = 0.0001). War/ Holocaust trauma and childhood trauma were also associated with increased dementia risk (HR = 1.28 (95% CI 1.01–1.63, P = 0.02) and HR = 1.76 (95% CI 1.17–2.64, P = 0.007) respectively).

**Conclusions:**

We have found an association between TLE and dementia risk. Future research exploring the dimensions of TLE and individual level factors are needed to better understand the relationship between TLE and dementia.

**Trial Registration:**

PROSPERO CRD42021253090.

**Supplementary Information:**

The online version contains supplementary material available at 10.1186/s12877-023-04287-1.

## Introduction

It is estimated that more than 150 million people will be living with dementia by 2050 [1]. Dementia impacts those with the condition and their loved ones. The care and treatment of those with dementia costs an estimated one trillion US dollars per year worldwide [2]. Several potentially modifiable risk factors have since been identified which, if addressed, could prevent dementia [2].

Studies have suggested that experiencing traumatic life events (TLE) may contribute towards dementia risk [3]. TLE has been defined as: actual or threatened death, significant injury or sexual violence. Events can be experience either first-hand or watching the event occur to others; learning of the violent or freak traumatic events that have occurred to a loved one; or repeated and/or severe subjection to unpleasant specifics of traumatic events e.g., law enforcement officers consistently handling cases of child abuse [4].

Trauma affects individuals immediately and the extreme stress that can be experienced as a result of trauma may have long-lasting consequences, including causing the brain to be more vulnerable to diseases, such as those which cause dementia. This is thought to be even more profound during critical points such as during brain development in childhood and as the brain declines in old age [5, 6]. Between 28 and 90% of adults in economically developed countries experience one or more TLE but only 3.9% go on to experience post-traumatic stress disorder (PTSD) [7].

Despite systematic reviews reporting a relationship between PTSD and dementia [8, 9], to our knowledge, none have examined the association between TLE and dementia risk and currently there are no meta-analyses. It is important to understand whether TLE themselves, and not only those that result in a diagnosis of PTSD, are a risk factor for dementia so that interventions to mitigate the risk can be developed to prevent or delay the onset of dementia in those who have experienced trauma. The current review aimed to address this gap in the literature by carrying out the first systematic review and meta-analysis of the relationship between TLE and all-cause dementia.

## Methods

### Protocol and guidance

The review was registered and published with PROSPERO (CRD42021253090) and followed preferred reporting items for systematic reviews and meta-analyses (PRISMA) guidelines [10].

### Inclusion and exclusion criteria

We included original research articles published in peer reviewed journals examining the association between TLE as defined by the American Psychiatric Association [4] and all cause dementia in individuals aged 60 and over. TLE included in the study were identified using the Life Events Checklist for DSM- 5 (LEC-5 [11]). Studies focussing on traumatic brain injury (TBI), toxic exposure, illness or physical injury were excluded from the review as, though the events may cause psychological trauma, the physical aspect of the events could lead to dementia via organic causes unrelated to the traumatic experience itself. We included studies which specified that dementia diagnosis was examined as an outcome, regardless of clinical criteria used for the diagnosis. Studies focussing on cognitive impairment and/or subjective cognitive decline were also excluded as these are not the same as a diagnosis of dementia. Where PTSD was the focus of the study the paper was excluded as this review was looking at a non-clinical population. To exclude early onset dementia, due to its different aetiology, all participants included in the study had to be 60 or over when diagnosed with dementia. Information concerning TLE was gathered from participants, their informants, or their medical records. All study designs, including case-control and cohort studies were included. To allow an accurate representation of those with dementia and to ensure that the review results are relevant to the real world, the study included papers relating to all-cause dementia, all severities, and all recruitment settings.

### Search strategy

We searched three databases - PsychINFO, Embase, and MEDLINE for terms relating to dementia, TLE, and risk and odds ratios from inception of each database until 29 May 2021 and updated on 20th April 2022. We combined terms for “dementia”, “risk” and different types of traumatic events using the AND operand. We placed no limits on language, study type or publication date. The search strategy is described in the supplementary material table S1.

### Selection process

All retrieved studies were exported to Covidence systematic review software [12] and duplicates were removed. All titles and abstracts were independently screened by two reviewers. ES screened all titles and abstracts while PL and TJ screened 50% each. Full text articles were then screened in the same way. Disagreements were discussed and resolved.

### Data extraction

Data were extracted by ES and independently reviewed by TJ. Information was extracted on the author, year of publication, country where the study was conducted, study design, number of participants and age at baseline, percentage gender split, population and recruitment of participants, length of follow up, assessment of TLE, type of trauma experienced, assessment of dementia, effect estimate, and variables adjusted for. Adjusted odds or hazard ratios were extracted where possible. Authors were contacted to request any missing information and odds ratios were converted into hazard ratios using published formulae [13] to allow for comparison.

### Quality assessment and risk of bias

Publication bias was assessed via a funnel plot performed on Review Manager version 5.4 for Mac [14]. A quality assessment of the studies was carried out independently by two reviewers, ES and TJ, using the Newcastle–Ottawa Scale developed to evaluate non-randomised epidemiological studies (NOS [15]). Different criteria were employed for case-control and cohort studies. The NOS contains three domains (selection, comparability, outcome/exposure) and operates on a star system with 9 stars being the maximum available, and a maximum of 4, 2, and 3 stars within each domain respectively. For more information on scoring see the supplementary material table S3. In order for a study to be classified as good it was required to have ≥ 3 stars in the selection domain, ≥ 1 star in the comparability domain and ≥ 3 stars in relation to outcome/ exposure. Fair studies were those with 2 stars in the selection domain but the same requirements as for good in the comparability and outcome/ exposure domains. Poor studies were those with ≥ 1 star in the selection domain and ≥ 0 stars in the two remaining domains. The outcome of the quality assessment was noted on a standardised table to allow for comparison. In the case of disagreement the two reviewers reached an agreement via discussion.

### Meta-analysis

We used RevMan software version 5.4 to meta-analyse studies using a random-effects model to obtain a pooled risk estimate for studies. We measured heterogeneity by the *I*^2^-statistic. We meta-analysed all studies and then conducted sub-analyses, grouping by type of trauma as long as there were at least two studies in each group. We also conducted sensitivity analyses, including only studies which reported hazard ratios and then only studies which were scored as being of high quality.

## Results

### Study selection

**Fig. 1:**
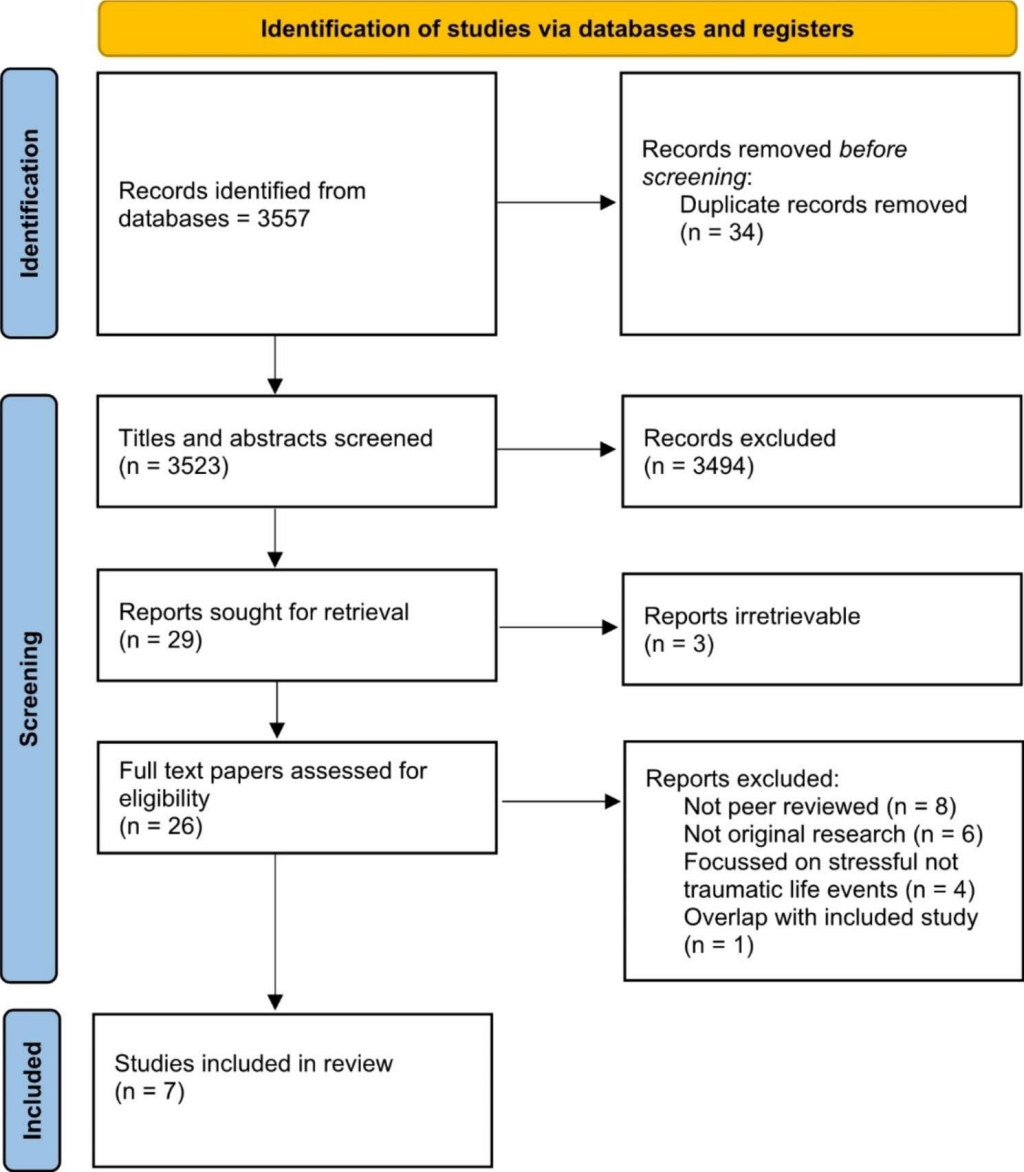
PRISMA flow diagram

The initial search retrieved 3,557 studies. After removal of duplicates, the titles and abstracts of 3,523 studies were screened, resulting in 29 papers being selected for full text screening. Three of these were not retrievable and 19 were excluded as they did not meet inclusion criteria (see Fig. 1 and supplementary table S2). Eight studies met the eligibility criteria, however, only seven of these were included in the meta-analysis as one [16] study used the same data set as another included study [3].Of these two studies we included the study which presented all data and did not stratify by social capital [3]. This process is shown in the PRISMA flow diagram (Fig. 1). Studies excluded from the review at the full text stage were coded with the reason for their exclusion and are set out in the supplementary materials (table S2). At full text stage there was an interrater agreement of 82.14% (κ = 0.58, 95% CI -1.17 to 2.33).

### Study characteristics

Of the seven studies included in the review, three were prospective cohort studies [3, 17, 18], three were retrospective cohort studies [19–21] and one study had a cross-sectional design [22]. The review included various forms of TLE: childhood trauma (two studies [3, 22], the Holocaust (two studies [19, 21] and one study each for intimate partner violence [17], Prisoner of War (POW [20] and non-specific TLE [18]. Mean follow up times ranged from 2 to 37 years (median 9.5 years).

The sample sizes ranged from 336^22^ to 182,879^20^, (median = 14,922) with data from 276,570 participants (50,863 female, 42,828 male and 182,879 unspecified gender) being used in total. The mean age of participants ranged from 50.3 to 77.0 years at baseline and none of the studies reported diagnosing early onset dementia. Three studies were from an Aboriginal and Torres Strait Islander population [22], the Civil Service in Israel [21], and with a veteran population in the USA [20]. Four studies used participants from the general population [3, 17–19] in Japan, Australia, France, and Israel respectively. There was a range of gender splits in this review: four studies included both genders [3, 18, 19, 22], one study focussed entirely on men [21], one focussed only on women [17] and one paper did not specify the gender composition [20]. However, this last study involved a veteran population and may have involved the assumption of a male cohort. The range of percentage female participants in studies using both genders was 53.3 to 59.8 [3, 22].

Exposure to traumatic events was ascertained through questionnaire, [3, 17, 18, 22] medical records [20], interview [21] or registration with Holocaust survivors authority [19]. Dementia diagnosis was determined through clinical assessment using well established clinical criteria and validated instruments in three studies [18, 21, 22]. Two studies determined dementia status through clinical records using the International Classification of Disease framework [19, 20] and the remaining two used insurance records but did not specify what diagnostic framework was used [3, 17].

Six studies [3, 17–20, 22] were deemed high quality and one [21] fair quality (indicating an increased risk of bias) (see supplementary material tables S4 and S5). Four studies had statistically significant findings indicating that TLE increased risk for dementia [3, 19, 20, 22] and three studies reported no increased risk [17, 18, 21]. Of the studies showing that exposure to traumatic events increased the risk of dementia, two were retrospective cohort studies, one was a prospective study and one had a cross-sectional design. Of those showing no association between TLE and dementia, two were prospective cohort studies and one was retrospective. All studies adjusted estimates for age, sex (if both sexes included) and some measure of socioeconomic status or education with most studies also adjusting for a range of health conditions. Characteristics of the included studies are displayed in Table 1.

**Table 1:** Details of included studies

Author (year) country	Study Type Recruitment	Sample at baseline (female %)	Type of Trauma	Trauma assessment/ diagnostic criteria	Dementia assessment/ diagnostic criteria	Follow up years	Effect estimate	Variables adjusted for	Quality Rating
Cations et al., (2020) Australia	Prospective Cohort Australian Medicare Database	Female general population N = 12,432 (100) Mean age = 72.6	Intimate Partner Violence	Self-report Questionnaires	Insurance Records Self-report Questionnaires Does not specify framework	21	HR: 1.02, 95% CI 0.89–1.17	Competing risk of mortality; age; SES; BMI; psychological well-being; physical functioning; social support	7
Kodesh et al., (2019) Israel	Retrospective Cohort Meuhedet Healthcare Services Data Registry	General Population N = 51,752 (54.0) Mean age = 60.4	Holocaust Survival	Holocaust Survivors Rights Authority at the Ministry of Finance	Meuhedet Dementia registry ICD 9 ICD 10	10	h: 1.21, 95% CI 1.15–1.28	Age; sex; SES; obesity; diabetes; cancer; delirium; vitamin deficiencies; concussion; migraine; depression; PTSD; sleep disorders; pain disorders; schizophrenia	8
Meziab et al., (2014) USA	Retrospective Cohort Veterans Health Administration National Patient Care Database	Veterans N = 182, 879 Gender not specified Mean age: POW = 77 Mean age non-POW = 68	Prisoner of War (POW)	Medical records	Medical records ICD 9 ICD 10	9	h: 1.61, 95% CI 1.30–1.98	Competing risk of mortality; age; education; income; hypertension; diabetes; myocardial infarction; cerebrovascular disease; peripheral vascular disease; chronic pulmonary disease; renal disease; major depressive disorder; period of service	8
Nilaweera et al., (2020) France	Prospective Cohort Electoral rolls of the Montpellier district	General population N = 1700 (59.0) Mean age = 72.6	Non-Specific Traumatic Event	French self-report version of the Watson’s PTSD Inventory DSM IV	MMSE Isaac’s Set Test Benton’s Visual Retention Test Trail Making Tests A and B DSM-IV NINCDS/ADRDA	14	HR:1.06, 95% CI 0.77–1.58	Age; sex; education; global cognition; morbidity; depression	9
Radford et al., (2019) Australia	Cross-Sectional Snowball sampling 62% of the total Aboriginal and Torres Strait Islander population aged > 60 years Five catchment areas in New South Wales	Aboriginal and Torres Strait Islander Australians N = 336 (59.8) Mean age = 66.7	Childhood trauma	Childhood Trauma Questionnaire	Comprehensive medical assessment NINCDS/ADRDA DSM-IV	2	OR:1.63, 95% CI 1.11–2.39	Age; sex; education; urban regional location	8
Ravona-Springer et al., (2011) Israel	Retrospective Cohort Israeli Ischemic Heart Disease (IIHD) Project Population based study Stratified sampling of tenured civil servants and municipal employees 86.2% participation rate	Male civil servants and municipal employees N = 10,059 (0.0) Mean age = 50.3	Holocaust/ Concentration Camp Survival	Interview	Telephone Interview for Cognitive Status Dementia Questionnaire MMSE Global Deterioration Scale Hachinski Ischemic Scale DSM-IV	Range = 36 to 37	OR: 0.93, 95% CI 0.58–1.49	Age; SES; diabetes; coronary heart disease; lung disease; kidney disease; height	7
Tani et al., (2020) Japan	Prospective Cohort Study The Japan Gerontological Evaluation Study Cohort Population Based study 24 municipalities throughout Japan 71% response rate	General population N = 17,412 (53.3) Mean age = 73.5	Childhood trauma	Adverse childhood experience questionnaire for older Japanese people	Japan’s public long-term care insurance registry Standardized in-home assessment and medical examination Does not specify framework	Mean = 3.2 Range = 2.4 to 3.3	h:1.78, 95% CI 1.15–2.75 (3 or more childhood adverse experiences)	Age; sex; childhood economic hardship; height; education; hypertension; diabetes; stroke; heart disease; annual income; longest occupation; marital status; frequency of meeting friends; social participation; employment status; smoking; BMI; depressive symptoms; hearing loss	7

**OR = odds ratio, HR = hazard ratio, 95% CI = 95% confidence interval, NINCDS/ADRDA = National Institute of Neurological and Communicative Disorders and Stroke and the Alzheimer’s Association criteria**

### Trauma and dementia

**Fig. 2:**
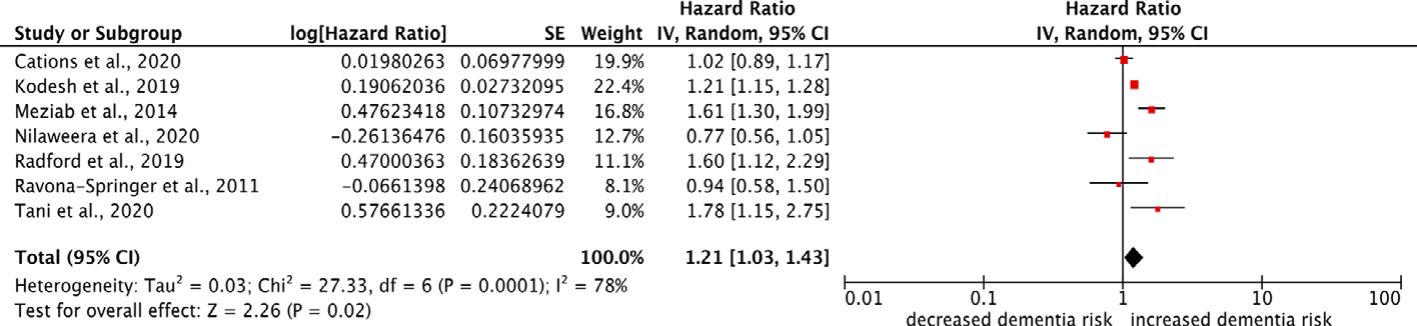
Meta-analysis of all included studies

**Fig. 3:**
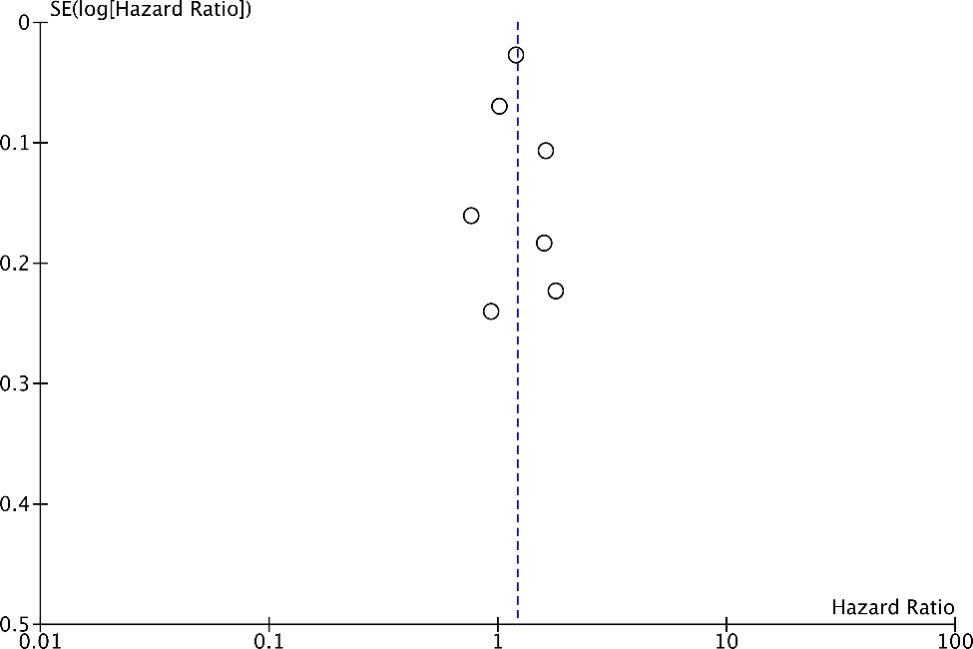
Funnel plot to assess publication bias

Pooled results from the seven included studies demonstrated that TLE increased the risk of all cause dementia (pooled HR = 1.21, 95% CI 1.03–1.43, P = 0.02; N = 276,570; median follow-up 9.5 years). This is shown in Fig. 2. There was significant heterogeneity (I^2^ = 78%). We generated a funnel plot (Fig. 3) which indicated no publication bias [23] (Egger’s test p = 0.4991).

### War/ holocaust trauma and dementia

**Fig. 4:**
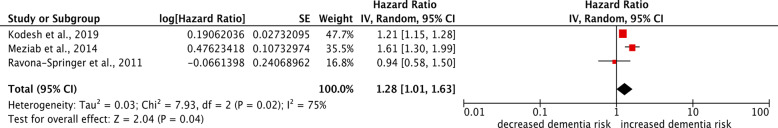
Meta-analysis of studies looking at war/Holocaust related trauma

Pooled results from three studies [19–21] showed that war and holocaust related TLE increased the risk of all cause dementia (pooled HR = 1.28, 95% CI 1.01–1.63, P = 0.04, N = 244,890; median follow-up 10 years) (Fig. 4). There was significant heterogeneity (I^2^ = 75%).

### Childhood trauma and risk of dementia

**Fig. 5:**
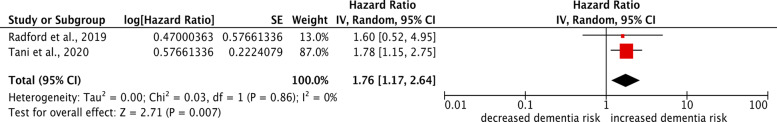
Meta-analysis of studies looking at childhood trauma

Pooled results from two studies [3, 22] showed that childhood trauma increased risk of all-cause dementia (pooled HR = 1.76, 95% CI 1.17–2.64, P = 0.007; N = 17,748, median follow-up 2.6 years (Fig. 5). There was no significant heterogeneity (I^2^ = 0%).

### Sensitivity analyses

Pooled results including only studies that reported hazard ratios gave an HR of 1.30 (95% CI 1.09–1.51). Meta-analysis including only studies which were rated as good quality gave a pooled HR of 1.34 (95% CI 1.14–1.54)(See supplementary figures S4 and S5).

## Discussion

We have systematically searched and reviewed the literature to identify studies examining the association between TLE and risk of dementia. To our knowledge, this is the first systematic review assessing the relationship between TLE and risk of dementia. This review found that TLE are associated with an increased risk of dementia. Sub-types of trauma occurring in childhood or in war also increase dementia risk though this conclusion is based on fewer studies. Gunak et al. [9] established an association between PTSD and risk for all-cause dementia. While we excluded studies that directly investigated PTSD it is likely that TLE can cause subclinical symptoms [24]. The mechanisms by which PTSD and TLE increase the risk of developing dementia are not fully understood but are likely to be similar. Altered activity in psychological (including repetitive negative thinking) and neurological pathways (including the hypothalamic-pituitary-adrenal axis), oxidative stress and a reduction in volume of the hippocampi as a result of trauma may play a part in increasing dementia risk [25, 26]. Recognising that PTSD and negative sequelae of TLE are potentially modifiable risks for dementia and understanding the mechanisms by which they increase vulnerability to dementia may allow the creation of interventions to specifically target these mechanisms and reduce the effect of trauma (and PTSD) on dementia.

Strengths of this review include its thorough and inclusive search strategy, independent screening by two reviewers at all stages and rigorous inclusion and exclusion criteria. The large sample size, which included outcomes of 276,570 participants and long follow up period are further strengths. However, the current review is not without its limitations. Relatively few studies were included - seven in total – and sub-types of trauma had even fewer studies included. For this reason we were unable to do many of the planned sub-groupings as outlined in our registered protocol. TLE often co-occur with other known risk factors of dementia (e.g. traumatic brain injury) [2].

Often psychological trauma is accompanied by physical harm e.g. in the case of Cations et al. [17], which investigated intimate partner violence. Accordingly, it is difficult to establish whether it is the psychological trauma resulting from the TLE that increases dementia risk, the physical injuries resulting from the event, or a combination of both. Three studies included in the review were retrospective cohort studies and one was a case control study which may have introduced selection bias and recall bias into the findings although the findings did not indicate that there was a systematic difference in findings between retrospective and prospective studies. Additionally, unlike other potential risk factors for dementia, reverse causation is unlikely to account for findings. In studies using self-report, trauma may be under reported for a number of reasons e.g. in Cations et al. [17], women may have under reported abuse because they did not recognise the behaviour as abusive, or because of shame, not feeling safe to report or for some other reason. We do not know the severity, chronicity or, in many cases, the age at which exposure to TLE occurred nor are we able to know the physical or psychological state of individuals before or after exposure to TLE so cannot judge how these factors influence dementia risk. Survival bias may also have been a factor to account for our findings. Individuals who experienced the most severe TLE may be underrepresented in older cohorts as premature death is linked to stress and trauma [27]. Conversely, those who survive into older age after experiencing trauma may also have physical advantages relating to ageing that may protect against dementia. The types of TLE experienced in these reviews varied greatly and it is possible that different types of TLE have different effects on dementia risk. Heterogeneity of studies in the meta-analysis was high (> 75% [28]) when all studies were grouped and in the prisoner of war/ Holocaust analysis but not in the childhood trauma analysis. It is likely that both methodological and clinical differences contributed to heterogeneity as there was variation in gender breakdown, setting, ascertainment of TLE and measurement of dementia.

Future studies are required to investigate the impact of TLE related factors such as chronicity and severity, and individual factors such as age on dementia risk and whether TLE is related to particular dementia subtypes. Further research should also address the mechanisms underpinning the relationship between TLE and dementia risk and focus on creating and assessing preventative interventions and treatments for dementia targeting the experience of trauma following TLE.

### Electronic supplementary material

Below is the link to the electronic supplementary material.

Supplementary Material 1

## Data Availability

The datasets used and/or analysed during the current study available from the corresponding author on reasonable request.
