# Supplementary material for: Traumatic life events and risk for dementia: a systematic review and meta-analysis
Source: BMC Geriatr. 2023 Sep 22;23:587. doi: 10.1186/s12877-023-04287-1 (PMC10517510; doi:10.1186/s12877-023-04287-1)

Supplementary Materials

Table S1

1.	(Dementia* or Alzheim* or AD or VAD).ab,kw,ot,ti.
2.	(Risk or odds or hazard).ab,kw,ot,ti.
3.	(trauma* or violen* or assault* or accident* or war or combat or natural disaster or terrorism or fire or explosion).ab,kw,ot,ti
4.	1 and 2 and 3
5.	limit 4 to humans
6.	remove duplicates from 5

Table S2
Excluded studies with reasons

Study	Reasons of exclusion
Ahmed et al., (2009)	Not peer reviewed- symposium abstract
Aliseychik et al., (2018)	Not original research- review
Barnes et al., (2013)	Not peer reviewed- symposium abstract
Bidzan & Ussorowska, (1996)	Contacted author received no response
Broe et al., (2016)	Not peer reviewed- symposium abstract
Broe, (2011)	Not peer reviewed- symposium abstract
Donley et al., (2018)	Included stressful life events
Eagle et al., (2022)	Not original research- commentary
Folnegovic-Smalc et al., (1997)	Contacted author received no response
Fujiwara et al. (2015)	Not peer reviewed- symposium abstract
Handforth & Parker, (2018)	Included stressful life events
Hoeijmakers et al., (2018)	Not original research- review
Johansson, (2014)	Not original research- review
Marchant & Howard, (2015)	Not original research- review
Nilaweera et al., (2019)	Not original research- review
Radford et al., (2018)	Not peer reviewed- symposium abstract
Reich, (2011)	Included stressful life events
Reichmann & Holzer, (2016)	Not original research- review
Shen et al., (1994)	Contacted author received no response
Stebbins, (2020)	Not peer reviewed- dissertation
Tani et al., (2021)	Used the same data set as an included study
Tsolaki et al., (2010)	Included stressful life events

Figure S1

Funnel Plot of Included Studies

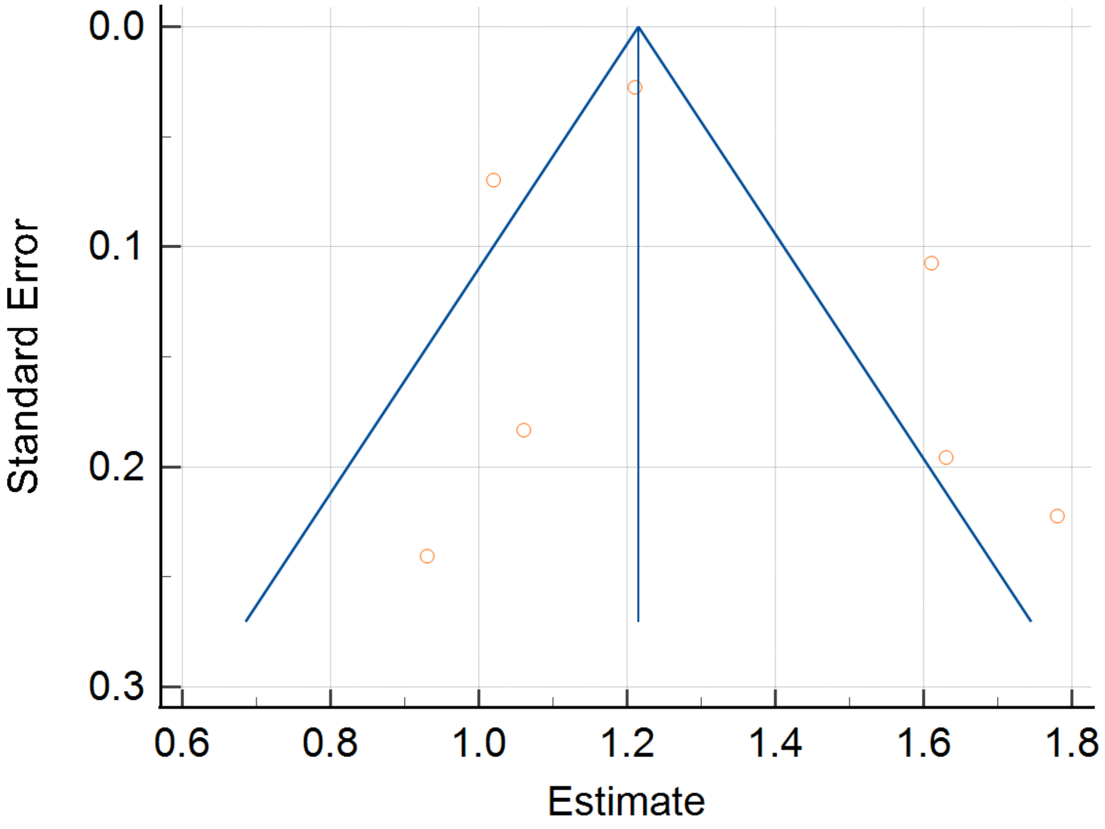

Figure S2

Funnel plot of studies relating to war/Holocaust

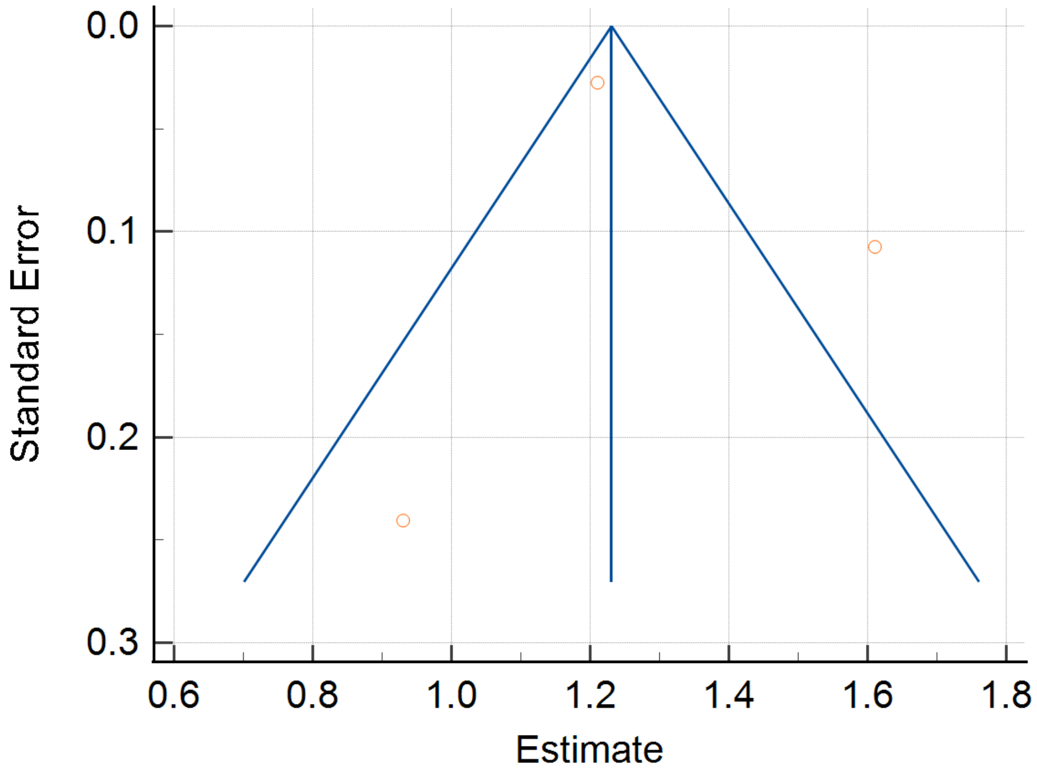

Figure S3
Funnel plot of studies relating to childhood trauma

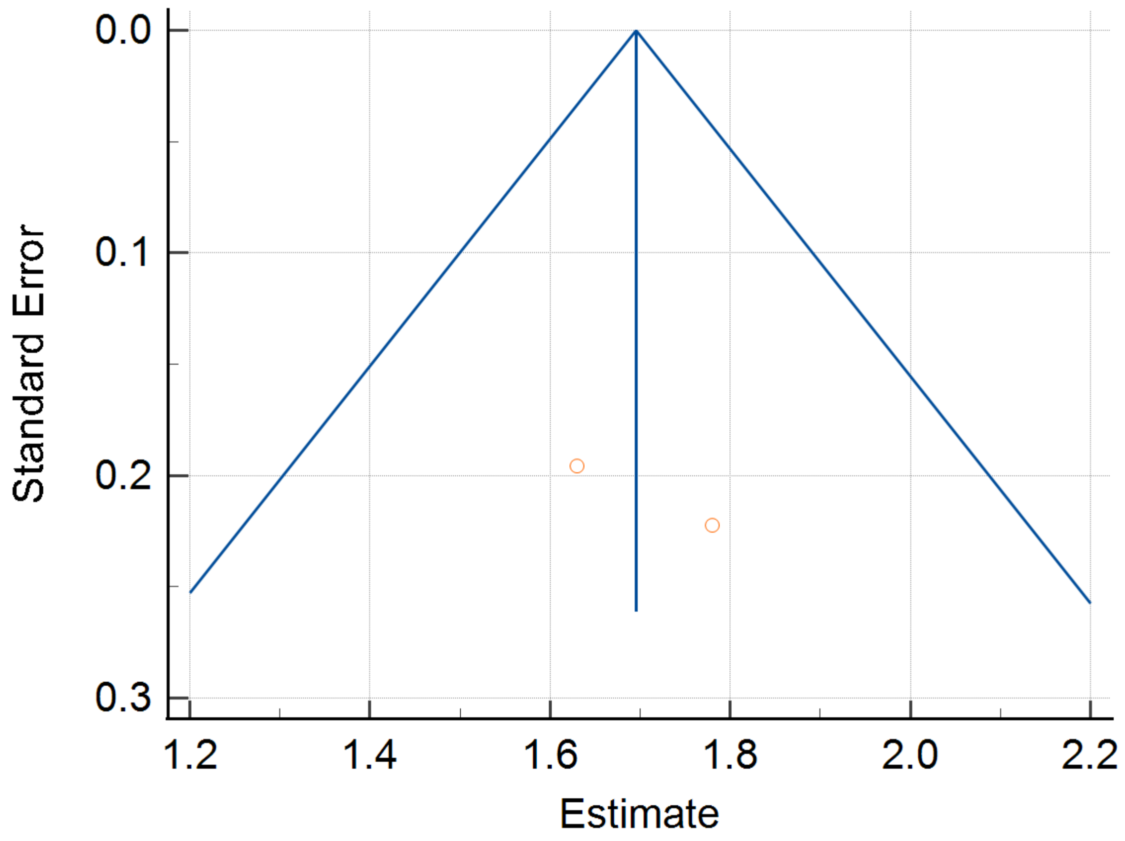

Table S3

Modified Newcastle-Ottawa Scoring scale assessing study quality

**Case Control**	**Cohort**
Selection	Selection
1. Is the case definition adequate?<br>a) yes, with independent validation (0 stars)<br>b) yes, eg record linkage or based on self-reports (0 stars)<br>c) no description (0 stars)	1. Representativeness of the exposed cohort<br>a) truly representative of the average specified population in the community (1 star)<br>b) somewhat representative of the average specified population in the community (1 star)<br>c) selected group of users e.g. nurses, volunteers (0 stars)<br>d) no description of the derivation of the cohort (0 stars)
2. Representativeness of the cases<br>a) consecutive or obviously representative series of cases (1 star)<br>b) potential for selection biases or not stated (0 stars)	2. Selection of the non-exposed cohort<br>a) drawn from the same community as the exposed cohort (1 star)<br>b) drawn from a different source (0 stars)<br>c) no description of the derivation of the non-exposed cohort (no stars)
3. Selection of Controls<br>a) community controls (1 star)<br>b) hospital controls (no star)<br>c) no description (0 stars)	3. Ascertainment of exposure<br>a) secure record (eg surgical records) (1 star)<br>b) structured interview (1 star)<br>c) written self-report (0 stars)<br>d) no description (0 stars)
4. Definition of Controls<br>a) no history of trauma (endpoint) (1 star)<br>b) no description of source (0 stars)	4. Demonstration that outcome of interest was not present at start of study<br>a) yes (1 star)<br>b) no (0 stars)
Comparability	Comparability
1. Comparability of cases and controls on the basis of the design or analysis<br>a) study controls for age (1 star)<br>b) study controls for any additional factor (1 star)	1. Comparability of cohorts on the basis of the design or analysis<br>a) study controls for age (1 star)<br>b) study controls for sex (1 star)
Exposure	Outcome
1. Ascertainment of exposure<br>a) secure record (eg surgical records) (1 star)<br>b) structured interview where blind to case/control status (1 star)<br>c) interview not blinded to case/control status (0 stars)<br>d) written self-report or medical record only (0 stars)<br>e) no description (0 stars)	1. Assessment of outcome<br>a) independent blind assessment (1 star)<br>b) record linkage (1 star)<br>c) self-report (0 stars)<br>d) no description (0 stars)
2. Same method of ascertainment for cases and controls<br>a) yes (1 star)<br>b) no (0 stars)	2. Was follow-up long enough for outcomes to occur<br>a) yes (greater than a year) (1 star)<br>b) no (0 stars)
3. Non-Response rate<br>a) same rate for both groups (1 star)<br>b) non respondents described (0 stars)<br>c) rate different and no designation (0 stars)	3. Adequacy of follow up of cohorts<br>a) complete follow up - all subjects accounted for (1 star)<br>b) subjects lost to follow up unlikely to introduce bias - small number lost - > 90 % follow up, or description provided of those lost) (1 star)<br>c) follow up rate < 90% (select an adequate %) and no description of those lost (0 stars)<br>d) no statement (0 stars)

Note: A study can be awarded a maximum of one star for each numbered item within the Selection and Outcome categories. A maximum of two stars can be given for Comparability Good: 3 or 4 points in selection domain AND 1 or 2 points in comparability domain AND 2 or 3 points in outcome domain; fair: 2 points in selection domain AND 1 or 2 points in comparability domain AND 2 or 3 points in outcome domain; poor: 0 or 1 point in selection domain OR 0 points in comparability domain OR 0 or 1 points in outcome domain;
†Modified for the systematic review

Table S4

Methodological quality of included cohort studies

Study Title	Selection				Comparability	Outcome			Overall Quality
	Representativeness	Control group	Ascertainment of exposure	Outcome not at baseline	Adjusted Covariates †	Assessment of outcome	Follow up length	Adequacy of follow up	
(Wells et al., 2000)	1	1	0	1	2	0	1	1	Good
Kodesh et al., 2019	1	1	1	1	2	1	1	0	Good
Meziab et al., 2014	0	1	1	1	2	1	1	1	Good
Nilaweera et al., 2020	1	1	1	1	2	1	1	1	Good
Ravona-Springer et al., 2011	0	1	0	1	2	1	1	1	Fair
Tani et al., 2020	1	1	0	1	2	1	1	0	Good
Tani et al., 2021	1	1	0	1	2	1	1	0	Good

Note. †Up to 2 points for adjusted covariates

Table S5

Methodological quality of included case control studies

Study Title	Selection				Comparability	Exposure			Overall Quality
	Case definition	Representativeness of cases	Selection of controls	Definition of controls	Adjusted Covariates †	Ascertainment of exposure	Ascertainment for cases and controls	Non-response rate	
Radford et al., 2019	1	1	1	1	2	0	1	1	Good

Note. †Up to 2 points for adjusted covariates

Figure S4

Forest plot of cohort studies

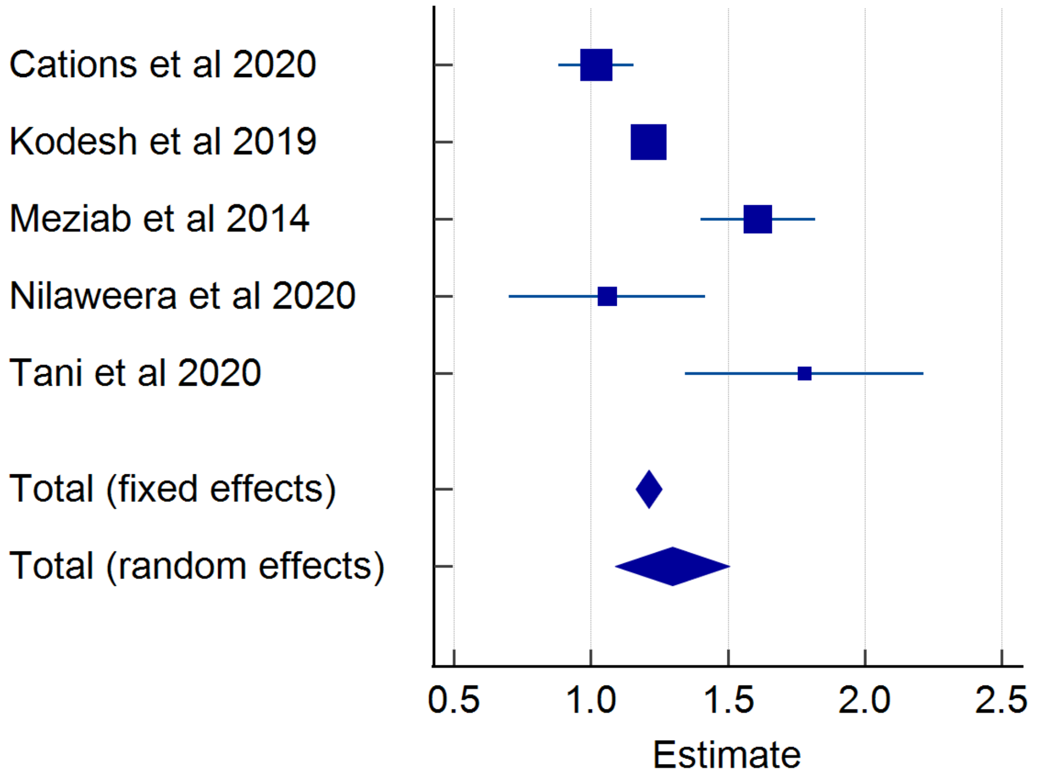

Figure S5

Forest plot of good quality studies

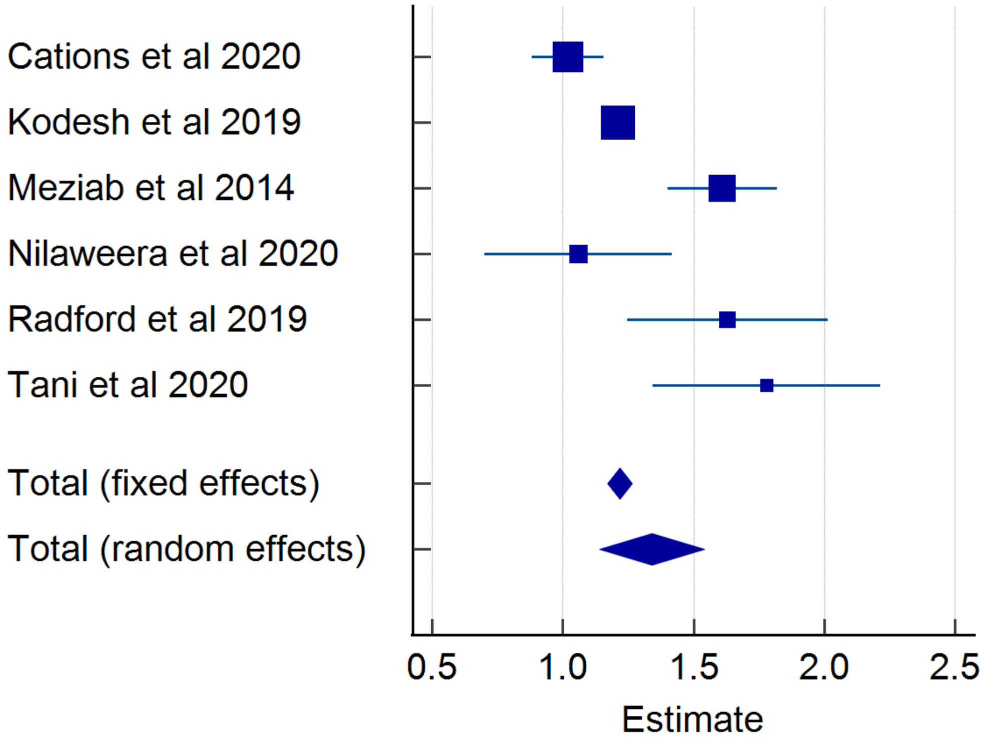

Supplement: Supplementary file 1 — Supplementary Material 1 [file 12877_2023_4287_MOESM1_ESM.pdf]
